# Elevated serum concentrations of GFAP in hereditary transthyretin amyloidosis since pre-symptomatic stages

**DOI:** 10.1007/s00415-025-13072-6

**Published:** 2025-04-15

**Authors:** Domenico Plantone, Marco Luigetti, Carlo Manco, Angela Romano, Luca Leonardi, Valeria Guglielmino, Francesca Forcina, Marco Ceccanti, Maurizio Inghilleri, Fiore Manganelli, Stefano Tozza, Maria Ausilia Sciarrone, Francesca Vitali, Andrea Sabino, Delia Righi, Angela Stufano, Maria Laura Stromillo, Nicola De Stefano, Paolo Calabresi, Guido Primiano

**Affiliations:** 1 Department of Medicine, Surgery and Neuroscience, University of Siena, Siena, Italy; 2 Dipartimento Di Neuroscienze, Organi Di Senso E Torace, Fondazione Policlinico Universitario Agostino Gemelli IRCCS, Rome, Italy; 3 Dipartimento Di Neuroscienze, Università Cattolica del Sacro Cuore, Rome, Italy; 4 Neuromuscular and Rare Disease Centre, Neurology Unit, Sant’Andrea Hospital, Rome, Italy; 5 Dipartimento Di Neuroscienze, Salute Mentale E Organi Di Senso (NESMOS), Sapienza Università Di Roma, Rome, Italy; 6 Dipartimento Di Neuroscienze Umane, Sapienza Università Di Roma, Rome, Italy; 7 IRCCS Neuromed, Pozzilli, Italy; 8 Department of Neuroscience, Reproductive and Odontostomatological Science, University of Naples ‘Federico II’, Naples, Italy; 9 Interdisciplinary Department of Medicine, University of Bari Aldo Moro, Bari, Italy

**Keywords:** Amyloidosis, Neurodegeneration, Glial fibrillary acidic protein, Biomarkers

## Abstract

**Background:**

Hereditary transthyretin amyloidosis (ATTRv) is a rare disorder caused by pathogenic *TTR* gene variants. Glial fibrillary acidic protein (GFAP) and neurofilament light chain (NfL) are potential biomarkers for astrocyte activation and neuroaxonal damage, respectively. This study investigates serum GFAP (sGFAP) and NfL (sNfL) levels in ATTRv patients, pre-symptomatic subjects, and healthy controls (HCs) to evaluate their utility as biomarkers of disease progression and CNS involvement.

**Methods:**

Our multicenter cross-sectional study included 111 ATTRv patients (56 symptomatic, 55 pre-symptomatic subjects) and 183 HCs. Serum levels of sGFAP and sNfL were measured using ultrasensitive immunoassays. The statistical comparisons were performed using ANCOVA models (age and sex adjusted), with correlations examined between serum biomarkers and disease severity (Neuropathy Impairment Score, NIS).

**Results:**

sGFAP levels were elevated in symptomatic (median: 238.35 pg/ml) and pre-symptomatic subjects (median: 105.50 pg/ml) *vs.* HCs (median: 75.5 pg/ml, *p* < 0.001). sNfL was elevated only in symptomatic patients (median: 43.68 pg/ml) compared to pre-symptomatic subjects (median: 9.36 pg/ml) and HCs (median: 7.54 pg/ml, *p* < 0.001). Both biomarkers correlated significantly with NIS, reflecting disease severity. Female HCs had higher sGFAP levels than males (median 88.6 pg/ml *vs.* 59.8 pg/ml; *p* 0.011).

**Conclusion:**

sGFAP and sNfL mark distinct ATTRv stages, with sGFAP indicating early preclinical changes and sNfL correlating with neurological progression. Sex differences in sGFAP levels among HCs suggest that sex should be considered as a covariate in biomarker analyses.

## Introduction

Hereditary transthyretin amyloidosis (ATTRv, v for variant), also known as familial amyloid polyneuropathy (FAP), is a severe, progressive, and multisystem disease caused by pathogenic variants in the *TTR* gene and is ultimately fatal if left untreated [1]. This autosomal-dominant neurogenetic disorder is rare, with an estimated global prevalence between 5500 and 38,500 cases [2]. It typically manifests in adulthood with variable penetrance and phenotypic heterogeneity, even among individuals harboring the same mutation [3].

Clinically, ATTRv manifestations include progressive sensori-motor axonal polyneuropathy, cardiomyopathy, and variable gastrointestinal, renal and ocular involvement. Central nervous system (CNS) manifestations, though sometimes under-recognized, are common and include meningeal and parenchymal involvement [4, 5]. Pathologically, TTR production in the choroid plexus remains unaffected by liver transplantation and other disease-modifying therapies due to their inability to cross the blood–brain barrier [6]. Consequently, amyloid accumulation in the CNS persists throughout the disease course.

Identifying reliable biomarkers for ATTRv is critical for early of diagnosing, monitoring disease progression, and assessing the effectiveness of currently available therapies. In this context, neurofilament light chain (NfL) has emerged as a suitable biomarker for detecting neuroaxonal damage [7, 8] and monitoring clinical progression [8–11] and therapeutic response [9, 10, 12–16].

Glial fibrillary acidic protein (GFAP), a type-III intermediate filament of astrocytes, is currently considered a biomarker of astrocyte damage and activation [17]. While GFAP is primarily associated with CNS astrocytes, it is also expressed in non-myelinating Schwann cells of the peripheral nervous system (PNS) and in enteric glial cells within the enteric nervous system (ENS) [18]. In the context of ATTRv, no data on serum GFAP (sGFAP) concentrations are currently available.

This study aims to investigate sGFAP and serum NfL (sNfL) levels in symptomatic and pre-symptomatic ATTRv patients and healthy controls (HCs), analyzing potential sex-related differences and their relationship with disease progression. Understanding GFAP dynamics may provide insights into ATTRv pathophysiology and guide improved diagnostic and therapeutic strategies.

## Methods

### Patients and healthy controls

This multicenter cross-sectional cohort study included subjects with confirmed pathogenic *TTR* variants, comprising both symptomatic ATTRv patients and pre-symptomatic subjects. The participants were recruited from Italian reference centers with extensive expertise in diagnosing and managing ATTRv. All enrolled symptomatic and pre-symptomatic subjects underwent comprehensive evaluations, including demographic, genetic, and neurological assessments conducted by expert neurologists, as detailed in a previous study [19]. The predicted age of onset of symptomatic disease (PADO) has been estimated for all 55 pre-symptomatic ATTRv subjects, considering the specific TTR gene mutation, the typical age of onset for that mutation and the age of onset in other members of the proband’s family, as previously recommended [20]. The difference of the age of the pre-symptomatic ATTRv subjects and the PADO was, therefore, calculated and named “Years from PADO”.

The control samples were collected from a cohort of HCs with no evidence of neurological, cardiac, renal, or autoimmune disorders. The study adhered to the principles outlined in the 1964 Declaration of Helsinki and its subsequent revisions. The study was approved by the Comitato Etico Territoriale Lazio Area 3 at the Fondazione Policlinico Agostino Gemelli IRCCS (protocol ID 5470).

### Serum sample collection

Peripheral blood was collected in additive-free vacutainers with separating gel, centrifuged at 3000 rpm for 10 min at room temperature, and the serum was stored in sterile polypropylene tubes at − 80 degrees Celsius.

### NfL and GFAP assay

sNfL and sGFAP levels were measured in ATTRv and HCs samples using the commercially available immunoassay kits for NfL and GFAP-SimoaTM assay Neurology 2-Plex B (GFAP, NfL) Assay Kit (Catalog #103520; Quanterix, Billerica, MA, USA). The assay was run on the semi-automated ultrasensitive SR-X Biomarker Detection System (Quanterix). Samples were diluted 1:4 and randomly distributed on 96-well plates. Quality control (QC) samples, provided with the kit, exhibit concentrations in the predefined range, and the coefficient of variance between plates was maintained below 10%. All samples were analyzed in a blinded manner using alphanumeric codes. The diagnostic codes were revealed only after QC-verified NfL and GFAP concentrations were reported to the database manager. The concentrations were measured in pg/ml and documented in the database. The analyses were conducted at the laboratory of the Centre for Precision Medicine and Translation of the University of Siena, Italy.

### Clinical assessment

All enrolled subjects underwent an extensive medical evaluation. For patients with evidence of polyneuropathy, disease severity was assessed using the Neuropathy Impairment Score (NIS) [21].

### Statistical analysis

Descriptive analyses (median and 25 th–75 th percentiles) are reported in Table 1**.** Normal distribution was assessed using the Shapiro–Wilk test and logarithmic transformations (Log10) were applied when the normality assumption was violated, following published studies [22]. Differences in sGFAP and sNfL levels within and between groups were evaluated using analysis of covariance (ANCOVA) models, adjusting for age and sex. A value of *p* < 0.05 was considered significant. ANCOVA was also used to perform a multivariate analysis considering age, gender, and disease severity assessed by NIS in ATTRv patients, and considering only age and gender in the whole ATTRv cohort. The correlations between sNfL, sGFAP, age, years from PADO (the latter in a partial correlation, considering age as covariate) and NIS clinical score were investigated using two-tailed Spearman's correlation. The analysis results and graphs were generated with Jamovi Software (The Jamovi Project, 2021).

**Table 1:** Detailed demographics of ATTRv patients, pre-symptomatic subjects and healthy controls (HCs), as well as TTR gene mutations, sNfL and sGFAP concentrations

	HCs	ATTR pre-symptomatic subjects	ATTR patients
Sex (F/tot)	109/183	28/55	13/56
Age (median, 25th–75th percentile)	50 (32–61)	47 (42,5-58)	71 (66,75-75)
Mutation (number of subjects, median age, number of female subjects)		V30M (30; 45 years; 16 F) F64L (14; 50.5 years; 6 F) I68L (3; 44 years; 3 F) V122I (3; 59 years; 0 F) E89Q (3; 47 years; 2 F) A109S (1; 45 years; 1 F) E62 K (1; 59 years; 0 F)	V30M (28; 69.5 years; 4 F) F64L (16; 74 years; 6 F) I68L (4; 73 years; 0 F) V122I (3; 70 years; 0 F) E89Q (2; 71.5 years; 2 F) A109S (1; 78 years; 0 F) A120S (1; 72 years; 1 F) V32R (1; 65 years; 0 F)
sGFAP levels (median, 25 th–75 th percentiles)	75.5 pg/ml, 43.7–125.0 pg/ml	105.50 pg/ml, 68.92–195.42 pg/ml	238.35 pg/ml, 138.99–342.88 pg/ml
sNfL levels (median, 25 th–75 th percentiles)	7.54 pg/ml, 5.59–10.80 pg/ml	9.36 pg/ml, 5.59–12.97 pg/ml	43.68 pg/ml, 25.99–63.32 pg/ml

## Results

### Cohort demographics and genetic mutations

This study enrolled 111 subjects with confirmed pathogenic *TTR* gene variants, referred to as the “ATTRv cohort”, with a median age of 64 years (25 th–75 th percentile: 47–72 years). The cohort included 41 females and 70 males, divided into 56 symptomatic patients, referred to as “ATTRv patients”, and 55 pre-symptomatic subjects. The study also included a cohort of 183 HCs with a median age of 50 years (25 th- 75 th percentile: 32–61 years), comprising 109 females and 74 males.

A non-parametric t-test (U-Mann–Whitney) revealed no significant age differences between males and females in either the ATTRv cohort or the HCs group. Within the ATTRv cohort, the following *TTR* gene mutations were identified: 58 cases had V30M, 30 had F64L, 7 had I68L, 6 had V122I, 5 had E89Q, 2 had A109S, 1 had V32R, 1 had A120S, and 1 had E62 K.

Detailed demographics of ATTRv patients, pre-symptomatic subjects and HCs, as well as *TTR* gene mutations, sNfL and sGFAP concentrations are provided in Table 1.

### sGFAP and sNfL levels across groups

**Fig. 1:**
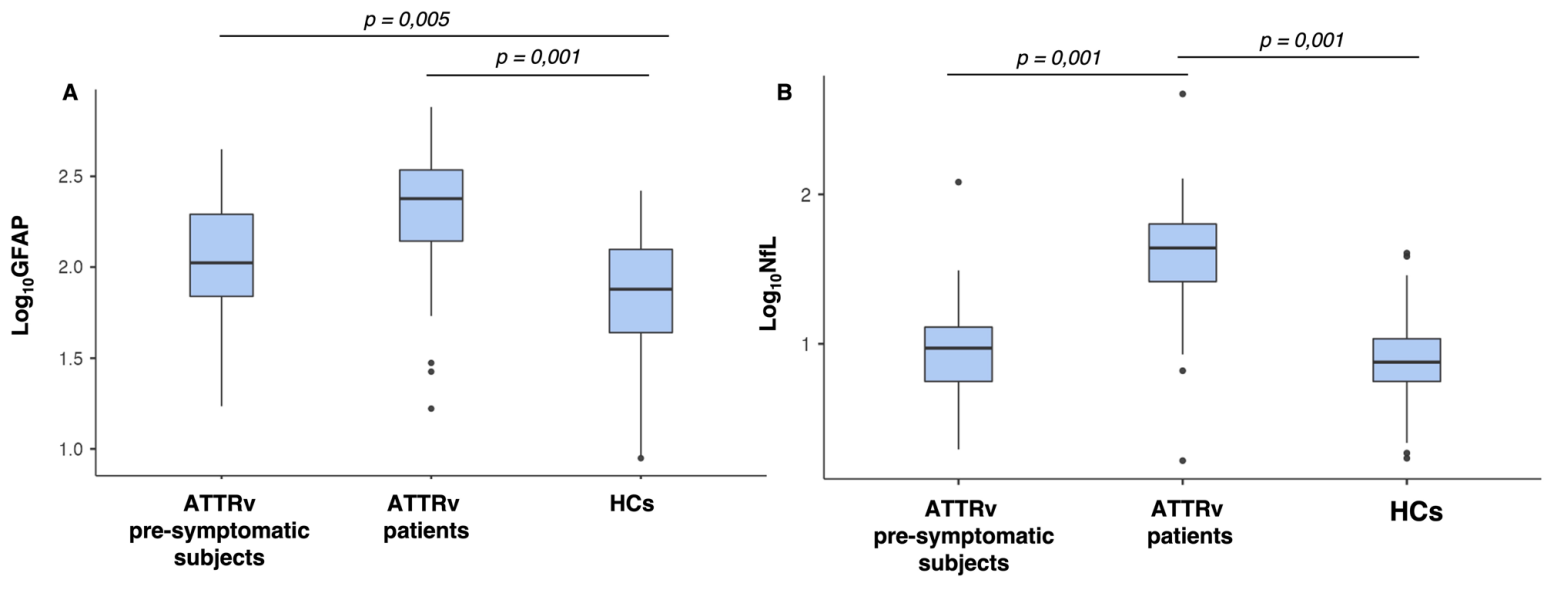
Comparison of sGFAP and sNfL levels (expressed as Log10-GFAP and Log10-NfL, respectively) across the three groups. **A** sGFAP levels were significantly higher in both ATTRv patients and pre-symptomatic subjects compared to healthy controls (HCs), **B** sNfL levels were significantly higher in ATTRv patients compared to both pre-symptomatic subjects and HCs

sGFAP levels were significantly higher in ATTRv patients and in pre-symptomatic subjects compared to HCs (*p* < 0.001 for both comparisons, Bonferroni-adjusted). No significant difference in sGFAP levels was observed between ATTRv patients and pre-symptomatic subjects (Fig. 1a).

sNfL levels were significantly higher in ATTRv patients compared to both pre-symptomatic subjects and HCs (*p* < 0.001 for both comparisons, Bonferroni-adjusted). No significant difference in sNfL was documented between pre-symptomatic subjects and HCs (Fig. 1b).

Due to the older age of ATTRv patients compared to HCs, we identified a subgroup of HCs which resulted age-matched with patients by selecting those older than 59 years. The resulted HCs population included 50 HCs (32 F) with a median age of 68.5 (25 th–75 th percentile 64–74 years; *p* = 0.41). By comparing this subgroup of HCs with ATTRv patients both sNfL and sGFAP concentrations resulted higher in the ATTRv patients compared to HCs (*p* < 0.001 for both; in the “old HCs” median sNfL 13.99 pg/ml; 25 - 75 th percentile 8.68–17.62; sGFAP 131.89 pg/ml; 25 - 75 th percentile 92.30–178.07).

**Fig. 2:**
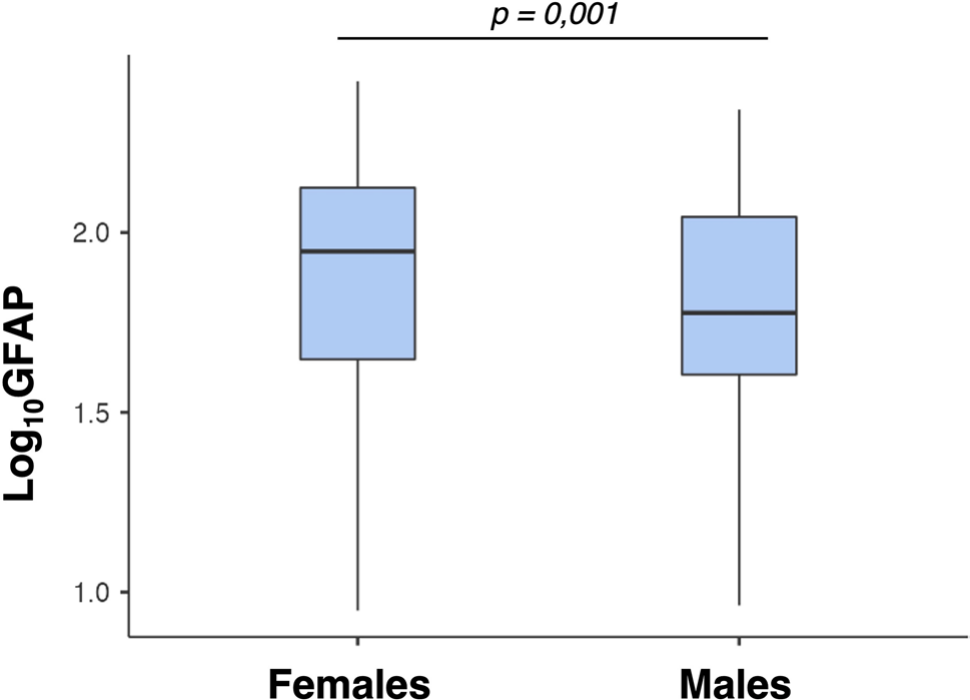
Comparison of sGFAP levels (expressed as Log10-GFAP) between females and males in the HCs cohort, demonstrating significantly higher sGFAP values in females compared to males

An ANCOVA analysis assessed the differences in sGFAP and sNfL levels between females and males in the HCs cohort. Female HCs had significantly higher sGFAP values than male HCs (*p* = 0.011, Bonferroni-adjusted) (Fig. 2). No significant sex differences were found in sNfL levels. The median concentrations, as well as 25 th and 75 th percentiles, of sGFAP and sNfL in female and male HCs, presymptomatic ATTRv subjects and ATTRv patients are reported in Table 2.

**Table 2:** Concentrations of serum glial fibrillary acidic protein (sGFAP) and neurofilament light chain (sNfL) in females and males in healthy controls (HCs), presymptomatic ATTRv subjects and ATTRv

	Female HCs	Male HCs	Female ATTR pre-symptomatic subjects	Male ATTR pre-symptomatic subjects	Female ATTR patients	Male ATTR patients
sGFAP levels (median, 25 th–75 th percentiles)	88.6 pg/ml, 44.4–133.0 pg/ml	59.8 pg/ml, 40.3–111.0 pg/ml	119.44 pg/ml, 79.59–205.99 pg/ml	89.05 pg/ml, 67.19–174.10 pg/ml	253.11 pg/ml, 176.60–346.27 pg/ml	209.70 pg/ml, 138.06–328.52 pg/ml
sNfL levels (median, 25 th–75 th percentiles)	7.5 pg/ml, 6.5–10.9 pg/ml	7.8 pg/ml, 5.5–10.3 pg/ml	9.02 pg/ml, 5.92–13.66 pg/ml	9.49 pg/ml, 5.59–12.21 pg/ml	43.70 pg/ml, 26.19–58.90 pg/ml	43.65 pg/ml, 25.80–70.19 pg/ml

In ATTRv patients and pre-symptomatic subjects, no significant sex-related differences in sNfL or sGFAP levels were observed. We also investigated the differences for gender in a multivariate analysis considering age, gender, and disease severity assessed by NIS in ATTRv patients and considering only age and gender in the whole ATTRv cohort. No significant difference was found in both analyses.

### Correlations

**Fig. 3:**
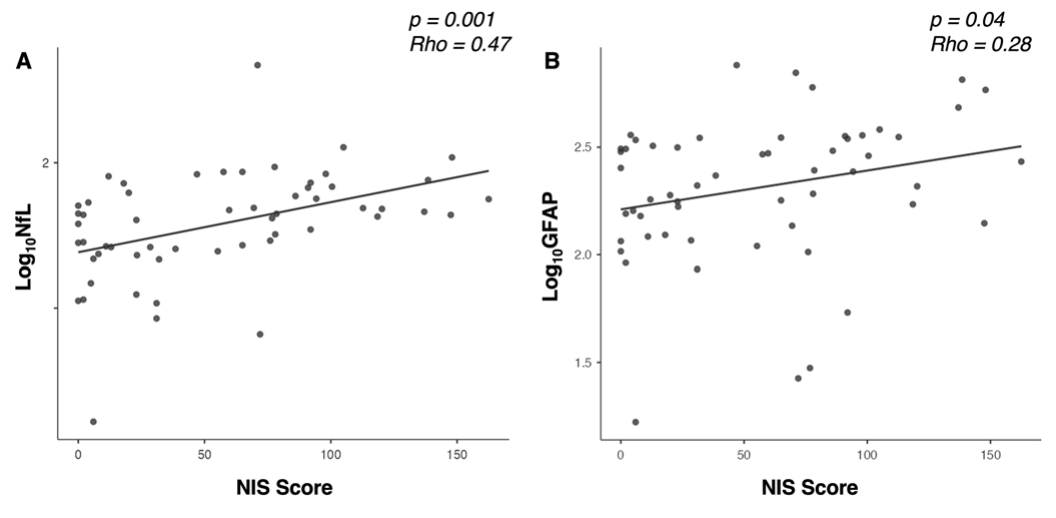
Correlation between sNfL and sGFAP levels (expressed as Log10-NfL and Log10-GFAP, respectively) with NIS scores, demonstrating a significant positive relationship between both biomarkers and clinical impairment

Spearman correlation analysis revealed significant relationships between biomarkers and clinical or demographic factors. Both Log_10_-NfL and Log_10_-GFAP showed a positive correlation with NIS scores (Rho 0.47, *p* < 0.001 and Rho 0.28, *p* = 0.04, respectively), as shown in Fig. 3.

In HCs group, age correlated significantly with both Log_10_-NfL (Rho 0.62, *p* < 0.001) and Log_10_-GFAP values (Rho 0.45, *p* < 0.001). When stratified the HCs group by sex, these correlations were confirmed for both females (Log_10_-NfL: Rho 0.61, *p* < 0.001; Log_10_-GFAP: Rho 0.48, *p* < 0.001) and males (Log_10_-NfL: Rho 0.66, *p* < 0.001; Log_10_-GFAP: Rho 0.48, *p* < 0.001).

In the ATTRv cohort, age correlated significantly with both Log_10_-NfL (Rho 0.65, *p* < 0.001) and Log_10_-GFAP (Rho 0.43, *p* < 0.001). Furthermore, a significant correlation was observed between Log_10_-NfL and Log_10_-GFAP within both the HCs group (Rho 0.53, *p* < 0.001) and the ATTRv cohort (Rho 0.68, *p* < 0.001).

In the 55 pre-symptomatic ATTRv subjects, we did not find any significant correlation between sNfL and sGFAP concentrations and years from PADO. On the contrary, when age was considered, it was positively correlated with sGFAP in the pre-symptomatic phase (Rho 0.25, *p* = 0.03).

## Discussion

This study highlights critical insights into the roles of sGFAP and sNFL in ATTRv. Our data documented increased sGFAP levels in both pre-symptomatic subjects and symptomatic patients compared to HCs, while elevated sNfL levels were found exclusively in symptomatic patients. This suggests that sGFAP elevation marks the disease from its preclinical stages, while sNfL increase corresponds with the presence of neurological manifestations. To the best of our knowledge, this study is the first to document significantly elevated sGFAP levels in both ATTRv patients and pre-symptomatic subjects.

The interpretation of GFAP elevation in the preclinical stage of the disease requires consideration of its origins within the nervous system. sGFAP, primarily recognized as a marker of astrocytic damage and activation within CNS, may also derive from the PNS and the ENS. However, the exact contribution of this production remains unclear. Investigating the different circulating isoforms of GFAP, a capability not yet available, could provide significant clarity on this aspect.

Existing evidence suggests that astrocytic activation in ATTRv begins as early as the pre-symptomatic stage [23], potentially driven by amyloid deposits in the CNS even before the onset of polyneuropathy. This activation follows the progressive stages of TTR-amyloidosis in the CNS, which increase in extent as the disease progresses [5]. Initially, TTR deposition is observed in the leptomeninges and subarachnoid meningeal vessels, with pronounced involvement in the brainstem and spinal cord. As the disease advances to the second stage, amyloid deposition extends to subpial cortical regions and becomes more frequent in perforating cortical vessels. In the final stage, the pathology spreads to subependymal regions and basal ganglia vessels near the ependymal lining. Notably, subpial TTR amyloid deposits are associated with astrocytosis, highlighting that astrocytic activation and damage are crucial features of ATTRv pathology and may significantly contribute to the observed elevation in serum GFAP concentrations.

Additionally, the elevation of sGFAP in pre-symptomatic subjects may also derive from the PNS and/or the ENS. GFAP expression in these systems is well-documented, particularly in Schwann cells and enteric glial cells [18]. GFAP is expressed in immature Schwann cells and subsequently downregulated in myelin-forming Schwann cells [24, 25], whereas non-myelinating Schwann cells retain GFAP expression and exhibit functional similarities to astrocytes. These non-myelinating Schwann cells could release GFAP into the bloodstream in case of dedifferentiation or mild (subclinical) nerve injury. Furthermore, glial cells in the ENS expressing GFAP may represent another potential source of sGFAP elevation. This sub-epithelial glia plays key trophic and neuromodulatory roles by supporting intestinal neurons and epithelial cells, regulating gut motility, and mediating neurotransmitter signaling. Given the high prevalence of gastrointestinal manifestations in ATTRv [26], it is plausible to hypothesize that early alterations in the ENS among TTR variants carriers could contribute to the observed sGFAP elevation.

The significance of the elevated sNfL levels in patients with ATTRv, which are absent in pre-symptomatic subjects, warrants specific consideration. sNfL is the most widely recognized biomarker of neuroaxonal damage, with its serum concentration reflecting contributions from both the CNS and the PNS [27, 28]. Notably, sNfL has been reliably shown to distinguish symptomatic patients with ATTRv polyneuropathy from pre-symptomatic subjects, a finding confirmed by our study [29]. Although sNfL is commonly utilized to monitor the clinical progression of patients with ATTRv polyneuropathy [30], definitive evidence attributing its entire concentration exclusively to the PNS is lacking [28]. Indeed, the pathological involvement of the CNS in ATTRv has been extensively documented and reported soon after the first description of the disease [31, 32]. Given the observed elevation of GFAP levels in our patient cohort, it is plausible to hypothesize that a portion of the sNfL may originate from the CNS, although the exact proportion remains undetermined. Future research should explore neurofilament isoforms with regional specificity to better localize and characterize the sources of sNfL elevation [28]. Interestingly, among these isoforms, peripherin seems a very promising biomarker of neuroaxonal damage involving the PNS and its serum concentrations have been already explored for this purpose [33].

Another important finding of our study is the higher sGFAP levels documented in female HCs compared to males. This aligns with previous research showing elevated plasma GFAP concentrations in cognitively unimpaired women compared to men [34]. Similar sex differences, characterized by higher peripheral blood GFAP levels in females, have also been described in Alzheimer's disease [34], Parkinson's disease [35], and in traumatic brain injury [36]. Sex-based variations in astrocyte number, differentiation, and function have been extensively documented [37–40]. Moreover, inflammation impacts the blood–brain barrier (BBB) differently between sexes. The differential expression of tight junction proteins and inflammatory markers between males and females underscores the importance of sex-specific mechanisms in neuroinflammation and BBB permeability [41]. These findings carry significant implications for the statistical analysis of scientific studies investigating sGFAP, as sex appears to be a significant covariate that must be considered for accurate interpretation of measurements.

The lack of significant differences in sGFAP levels between pre-symptomatic subjects and symptomatic patients might suggest that sGFAP serves more as a marker of disease presence rather than progression. In further support of this, no significant correlation was detected between sGFAP concentrations and years from PADO in the pre-symptomatic phase. However, several factors could contribute to this observation. One potential explanation is the sample size of our study, which might have limited the statistical power to detect subtle differences between these groups. Another possible contributing factor is the disparity in sex distribution between the pre-symptomatic group and the symptomatic patients, with a higher proportion of females in the former (51%) compared to the latter group (23%). Considering that sGFAP levels, as previously demonstrated, are generally higher in females, this sex imbalance may mask any potential differences attributable to disease progression. Future studies with larger and more balanced cohorts should explore these factors to better understand the dynamics of sGFAP levels across the stages of ATTRv.

A potential limitation of our study was the age disparity among the cohorts of HCs, presymptomatic subjects, and patients. Specifically, the patients were older than the subjects in the other cohorts. To address this limitation, we conducted all relevant analyses of sGFAP and sNfL levels while controlling for age as a covariate. Furthermore, we validated our findings by selecting a subgroup of older HCs who were age-matched with the ATTRv patients, thereby eliminating age-related differences.

Finally, the positive correlation between neurological impairment, as assessed by NIS, and sNfL and sGFAP concentrations supports their utility as biomarkers of disease severity. The association between sNfL levels and clinical disability has been previously demonstrated by our group using an alternative method for the assessment of sNfL [11]. Similarly, the relationship between sGFAP and clinical measures of disability has been described in peripheral neuropathy [42]. To the best of our knowledge, this study provides the first evidence of such a correlation in ATTRv patients.

## Conclusions

Our study demonstrates that sGFAP levels are significantly elevated in both symptomatic ATTRv patients and pre-symptomatic subjects compared to healthy controls, suggesting a possible contribution from the CNS, PNS, and ENS to this increase from the preclinical stages of the disease. This highlights the potential utility of sGFAP as a biomarker for early detection and monitoring of ATTRv, alongside sNfL, which serve as a marker of clinical involvement. Moreover, our findings suggest that GFAP elevation may stem from both central and peripheral nervous system involvement, reflecting the complex pathophysiology of ATTRv. Further research into GFAP isoforms and their specific contributions could provide valuable insights into disease mechanisms and biomarker refinement.

Moreover, the sex differences observed in sGFAP levels among healthy subjects suggest that sex should be considered as a covariate in analyses of this biomarker. These variations may reflect distinct differences in glial physiology between sexes. Ultimately, our study supports the use of both sGFAP and sNfL as biomarkers with distinct implications in ATTRv. Future longitudinal studies are needed to further characterize how these biomarker concentrations evolve throughout disease progression and in response to specific treatments.

## Data Availability

All data relevant to the study are included in the article.
